# Behavioral Assessment of Central Auditory Processing in Turner Syndrome

**DOI:** 10.1055/s-0043-1768141

**Published:** 2023-10-06

**Authors:** Adriana Fernandes Duarte dos Santos, Martha Marcela Matos Bazilio, Silvana Frota, Marilia Guimarães, Marcia Gonçalves Ribeiro

**Affiliations:** 1 Programa de Pós-Graduação em Clínica Médica, Faculdade de Medicina UFRJ, Rio de Janeiro, RJ, Brazil; 2 Department of Speech Therapy, Faculdade de Medicina UFRJ, Rio de Janeiro, RJ, Brazil; 3 Endocrinology Service, Faculdade de Medicina URFJ, Rio de Janeiro, RJ, Brazil; 4 Pediatrics Department, Faculdade de Medicina UFRJ, Rio de Janeiro, RJ, Brazil

**Keywords:** turner syndrome, gonadal dysgenesis, 45, X, hearing, auditory processing

## Abstract

**Introduction** Turner syndrome (TS) affects ∼ 1 in 2,500 live births. The presence of hearing alterations is one of the comorbidities found in this syndrome.

**Objective** The present study aimed to evaluate the central auditory abilities in TS and to associate the alterations found with the cytogenetic pattern of the syndrome.

**Methods** We included children and adults aged 9 to 39 years old, diagnosed with TS, with numerical or structural alterations of sex chromosomes in their karyotype. A battery of behavioral tests of central auditory processing (CAP) was performed, including a test within the modalities: monoaural low-redundancy, dichotic listening, binaural interaction, and temporal processing (resolution and ordering). We studied auditory skills in the total sample and in the sample stratified by age, divided into groups: G1 (9 to 13 years old), G2 (14 to 19 years old), and G3 (20 to 31 years old). For the association of the cytogenetic pattern, the division was T1 (chromosome monosomy X), and T2 (other TS cytogenetic patterns). Statistical analysis presented data expressed as median and interquartile range for numerical data and as frequency and percentage for categorical data.

**Results** We found alterations in four auditory skills in the three age groups, but there was a statistically significant difference between the age groups only in the *Gaps in Noise Test* (GIN) ( *p* -value = 0.009). Regarding karyotype, a greater number of alterations in the T1 cytogenetic pattern (chromosome monosomy X) was observed in four auditory skills, but without a statistically significant difference.

**Conclusion** The alterations found point to an impairment in CAP in TS.

## Introduction

Turner syndrome (TS) occurs in ∼ 1 in 2,500 live-born female infants. Its most frequent chromosomal constitution is the 45, X pattern, with the absence of the second sex chromosome. 1 Turner syndrome presents several characteristics, such as short height, gonadal dysgenesis, unusual facial features, webbed neck, low hairline at the back of the neck, broad chest with breast hypertelorism, renal and cardiovascular anomalies, sexual immaturity, swelling on the foot dorsum (in babies), aortic coarctation, hearing alterations, among other characteristics. 1 2

There are several reports of auditory function impairment in individuals diagnosed with TS. In relation to peripheral hearing, several types of alterations are described, such as conductive, mixed, or sensorineural hearing loss, mid-frequency sensorineural dip and a high-frequency sloping configuration. 3 4 5 6 7 8 9

It is very common for girls and women with TS to develop a progressive sensorineural hearing loss, like presbycusis, but at a much younger age and with much faster progression. 10 It is even recommended that women with TS undergo an annual hearing screening starting in childhood and for the rest of their lives, as hearing loss is not always promptly diagnosed. 10

However, when it comes to central auditory assessment, the number of studies is scarce, especially concerning Central Auditory Processing (CAP) assessment with TS. We found an international study that evaluated 30 women with TS, with two CAP tests. 11

Therefore, our study aimed to evaluate central auditory skills in patients with TS, as well as to associate the alterations found with the cytogenetic pattern of the syndrome.

## Method

This is a cross-sectional, descriptive, and observational research, with prospective and retrospective data collection, approved by the Research Ethics Committee of the Martagão Gesteira Institute of Pediatrics and Child Health (IPPMG/UFRJ), under number 1,864,065. The convenience sample consisted of patients diagnosed with TS referred to the Genetics Laboratory for cytogenetic diagnosis. The patients were referred from the Medical Genetics and Pediatric Endocrinology Services of the IPPMG/UFRJ and the Endocrinology Service of the Clementino Fraga Filho University Hospital (HUCFF/UFRJ).

We adopted the following inclusion criteria: children and adults, from 9 to 39 years old, diagnosed with Turner Syndrome, with numerical or structural changes in the sex chromosomes in the karyotype (lymphocyte culture), who had agreed to participate in the study with their consent or the consent of a parent or guardian.

We established minimum age limits for the analysis of normative values of central auditory processing tests, based on the maturation of central auditory areas. We also established maximum limits to avoid confounding bias with early presbycusis, which is reported in TS by some authors. 10 12 13

We excluded people with TS and intellectual disabilities, with other previously diagnosed associated genetic syndromes (by checking medical records), with a history of recurrent otitis, with type B or C tympanometry, with airway tonal thresholds > 25 dB (decibels) at any of the frequencies evaluated in pure tone audiometry. Pure tone audiometry and tympanometry were performed in the first phase of the present study.

The study was carried out in two locations: at the IPPMG/UFRJ Medical Genetics Service and the Audiology Division of the National Institute for the Education of the Deaf (DIAU/INES).

After the previous procedures necessary for sample selection, we performed a behavioral assessment of the CAP. For a comprehensive assessment of the central auditory pathways, we applied five behavioral tests of CAP, one to assess each auditory mechanism: a low-redundancy monaural test, a dichotic listening test, a binaural interaction test, and two temporal processing tests, one of them evaluating resolution and the other evaluating temporal ordering. The choice of tests was based on the recommendation of the Brazilian Academy of Audiology 14 15 with the following description:

Low redundancy monaural test: **Filtered Speech Test** . 16 Two lists with 25 phonetically balanced monosyllables were presented, one for each ear, at 40 dB SL, with frequency distortion (low-pass condition: cutoff at 400 Hz), where the participant was instructed to repeat each word heard. The test was considered altered when the number of correct answers was < 52% in at least 1 of the ears. The auditory skill assessed was auditory closure. 16

Dichotic listening test: **Dichotic Digit Test (DDT)** . 16 We presented four lists with 20 items each, formed by four of the following digits: four, five, seven, eight, and nine. Two digits were presented in each ear, simultaneously, at 50 dB SL.

The dichotic digit test was performed in the free attention stage (binaural integration) to reduce the test execution time. 17 The test was considered altered when the number of correct answers was < 95% in at least 1 of the ears. The auditory skill assessed was binaural integration. 15

Temporal order test: **Pitch Pattern Sequence Test (PPS)** . We used the test version proposed by Musiek et al., 18 in which three-tone sequences of two different frequencies were presented, both high (1,122 Hz) and low (880 Hz). The participant was instructed to listen carefully and inform the patterns (high/low), in the same order as heard, and inversions were considered errors. The test was performed at 50 dB SL, with binaural presentation. It was considered altered when the number of correct answers was < 72%. 19 The auditory skill assessed was temporal ordering. 16 18

Temporal resolution test: **Gaps in Noise Test (GIN).** 20 21 White noise segments were presented, with none or up to three periods of silence (gaps). The participant was instructed to press the audiometer's response button whenever she perceived a gap. The threshold was the minimum intensity that she hit in at least four of the six gaps presented, with the result expressed in milliseconds. The test was performed with monaural presentation and intensity of 50 dB SL. It was considered altered when the threshold obtained was > 6 milliseconds. 22 23 The auditory ability assessed was temporal resolution. 16 20

Binaural interaction test: **Masking Level Difference Test (MLD).** 17 24 25 26 Originally described by Hirsh. 27 We used a recording commercialized by Auditec of Saint Louis, Richard Wilson version. In this test, a pure pulsatile tone (500Hz) was presented in both ears, simultaneously with a narrowband masking noise, presented binaurally at 50 dB SL in three different conditions: SoNo (noise and pure tone presented in-phase), SπNo (pure tone presented in phase, with out-of-phase noise), and NT (noise without pure tone). The test was considered altered when the threshold was < 10 dB. 28 The auditory skill assessed was binaural interaction. 15

We chose to study the central auditory skills in the overall sample and then in the sample stratified by age to verify if there was a difference in performance in the behavioral tests in each group. As they are tasks with linguistic demand, some factors can interfere, such as neurological maturation and schooling.

The division was made into three groups, G1 age range from 9 to 13 years old, G2 age range from 14 to 19 years old, and G3 age range from 20 to 31 years old.

For the syndrome cytogenetic pattern association, we chose to divide the study sample into two groups: T1 with participants who presented monosomy of the X chromosome as a cytogenetic pattern, and T2 with participants who presented any other cytogenetic pattern of TS that was not monosomy of the X chromosome. This decision was made because there is a variety of other alterations, and this would stratify the sample in a way that would not bring us any answer to the proposed objectives.

We presented the observed data in form of tables, expressed by the median and interquartile range (Q1 and Q3) for numerical data and by the frequency and percentage for categorical data. We applied nonparametric methods, as the variables did not present a normal (Gaussian) distribution, according to the rejection of the normality hypothesis by the Shapiro-Wilk test. The criterion for determining significance adopted was the 95% level. Statistical analysis was processed using IBM SPSS Statistics for Windows version 26 (IBM Corp., Armonk, NY, USA).

## Results

Out of the 59 participants who underwent basic audiological assessment (audiometry and tympanometry), which was carried out in the first stage of the study, 38 had normal tonal thresholds at all frequencies, and tympanometric curves of type A, Ar or Ad, and were included in the CAP assessment survey. In the application of the CAP behavioral tests, only one participant was excluded, as she did not understand the instructions for performing the tests. After she left, we had a final study sample of 37 participants, between 9 to 31 years old, divided into 3 groups: G1, aged 9 to 13 years old ( *n* = 13); G2, aged 14 to 19 years old ( *n* = 12); and G3, aged 20 to 31 years old ( *n* = 12) ( Table 1 ).

**Table 1:** Results of central auditory processing assessment according to age group

Variable	9 to 13 ( *n* = 13)					14 to 19 ( *n* = 12)					20 to 31 ( *n* = 12)					*p-value*
	medial	median	IIQ			medial	median	IIQ			medial	median	IIQ			
FS – RE	71.4	72	64	–	76	73.7	82	59	–	84	81.0	84	72	–	91	0.12
FS – LE	78.5	80	70	–	86	79.0	84	72	–	88	84.3	82	80	–	91	0.35
DD – RE	95.8	95	93	–	100	96.3	100	91	–	100	95.8	100	91	–	100	0.91
DD – LE	90.4	95	85	–	100	95.4	95	91	–	100	94.6	100	91	–	100	0.35
MLD	9.2	10	7	–	12	9.2	10	8	–	12	11.0	12	8	–	14	0.35
GIN	5.6	** 5 ^ab^**	5	–	6	6.9	** 6 ^a^**	6	–	8	7.9	** 8 ^b^**	5.5	–	10	**0.013**
PPS	41.8	33	23	–	55	53.3	40	31	–	85	40.8	36.5	27	–	60	0.45
Number of alt tests	1.8	2	1	–	3	1.8	2	1	–	2.8	2.3	2	1.25	–	3	0.47

Abbreviations: FS-RE, Filtered Speech Test-right ear; FS-LE, Filteres Speech Test- left ear; DD-RE, Dichotic Digit Test -right ear; DD-RE, Dichotic Digit Test -left ear; MLD, Masking Level Difference Test; PPS, Pitch Pattern Sequence Test.

Data were expressed by medial, median and interquartile range and interquartile range (Q1-Q3) and compared by ANOVA Kruskal-Wallis test. According to the Dunn's Test for Multiple Comparisons, at level 5% we identified a significant difference between age groups 9 to 13 years old versus 14-19 years old , and between 9-13 years old versus 20-31 years old.

The responses obtained in each test of the PAC behavioral assessment were described in Table 1 , where we can observe a variability of responses in each test. There is a significant difference only in the GIN-AO between the age groups ( *p* = 0.013), with this difference not being < 0.05 in the other tests evaluated, between the three age groups.

Table 2 shows the results of each test, classified as normal and abnormal, divided by age group. The test that presented the greatest number of alterations was the PPS, with a result below expectations in all age groups evaluated. The test with the best performance was the Filtered Speech, where only one participant of the total sample showed alteration.

**Table 2:** Results of central auditory processing assessment with classification “normal” and “altered” according to age group

CAP tests	9-13		14-19		20-31		*p- value*
	*n*	%	*n*	%	*n*	%	
FS							
altered	0	0	1	8.3	0	0	0.65
normal	13	100	11	91.7	12	100	
DD							
altered	6	46.2	5	41.7	4	33.3	0.91
normal	7	53.8	7	58.3	8	66.7	
MLD							
altered	5	38.5	4	33.3	4	33.3	0.99
normal	8	61.5	8	66.7	8	66.7	
GIN							
altered	1	** 7.7 ^a^**	4	33.3	8	** 66.7 ^a^**	**0.009**
normal	12	92.3	8	66.7	4	33.3	
PPS							
altered	12	92.3	8	66.7	12	100	0.063
normal	1	7.7	4	33.3	0	0	

Abbreviations: FS, Filtered Speech Test; DD, Dichotic Digital Test; MLD, Masking Level Difference Test; PPS, Pitch Pattern Sequence Test

Data were expressed by frequency ( *n* ) and percentage (%) and compared by Fisher exact test. According to the Fisher exact test adjusted to level 1.7%, we identified a significant difference between age groups 9-13 years old versus 20-31 years old.

We correlated the results of each test with the cytogenetic pattern, where we observed a greater number of changes in participants with monosomy X (classified in our study as T1 cytogenetic pattern), and this group showed changes in four of the five tests applied in the study. The results are described in Table 3 .

**Table 3:** Results of central auditory processing assessment with classification “normal” and “altered” according to cytogenetic pattern

CAP Tests	Karyotype T1 (n = 15)		Karyotype T2 (n = 22)		*p value*
	n	%	n	%	
FS					
altered	0	0.0	1	5	0.59
normal	15	100.0	21	95	
DD					
altered	7	46.7	8	36.4	0.38
normal	8	53.3	14	63.6	
MLD					
altered	7	46.7	6	27.3	0.19
normal	8	53.3	16	72.7	
GIN					
altered	7	46.7	6	27.3	0.19
normal	8	53.3	16	72.7	
PPS					
altered	13	86.7	19	86	0.68
normal	2	13.3	3	14	

Data were expressed by frequency (n) and percentage (%) and compared by Fisher's exact test.

## Discussion

In the Scielo, PubMed, and Bireme databases, we did not find any study like ours, which assessed the central auditory pathway in its entirety with the application of a battery of behavioral tests. There was a study correlating TS with CAP behavioral tests, but it was not possible to correlate the findings, because the study only applied isolated tests, and there was no separation of the groups concerning the tonal audiometry result. 11 The authors aimed to correlate the results of peripheral assessment regarding sensorineural losses with central auditory responses in individuals with and without TS. 11 In our study, we only included participants with normal peripheral hearing.

What is described in the scientific literature in relation to central hearing in TS are studies investigating brainstem auditory evoked potentials (BAEP). 6 11 A study that evaluated 30 women aged 40 to 67 years old presented a significant difference between the control group and the group with TS concerning wave V latency. This demonstrates that participants with TS had higher wave V latencies, which may suggest a greater probability of central alteration in TS. 11

Another study with BAEP found alterations in 52% of the evaluated ears, with 24.5% of an isolated increase in wave I latency, 26.5% of an increase in all latencies, and 1 case with an isolated increase in wave V latency. All interpeak latencies were significantly increased in the group with TS in comparison with the control group and this could mean a greater tendency to central alterations in this population. 6

When analyzing the responses obtained in each test, with the respective measures of central tendency, we observed that there was a difference in the performance of each test, showing that in the sample of participants with TS there is a variability of responses in relation to central auditory skills ( Table 1 ). We also verified heterogeneity of responses in behavioral tests of other populations such as children with dyslexia and with learning disabilities. 29

In descending order of alterations found in our sample, the test in which we observed many altered results was the Pitch Pattern Sequence (PPS) test, in the three age groups evaluated ( Table 2 ). In group 1, aged from 9 to13 years old, > 90% had scores below average and in group 3, aged 20 to 31 years old, 100% of the participants had scores below average. The group with the lowest number of alterations was G2 (14 to 19 years old), with 2/3 of the participants presenting alterations in the test. The PPS also showed worse performance in children with dyslexia 30 and with attention deficit hyperactivity disorder (ADHD) 31 when compared with other CAP behavioral tests. The results were also altered in 73.33% of the sample of children with reading and writing disabilities, even using another version of the test (Taborga). 29

Following the analysis of our research, in the Dichotic Digits test, 15 participants presented altered results (40% of the sample), with homogeneous answers in each age group, with a decrease in right answers as age increased, but without a statistically significant difference ( Table 2 ). The performance of the dichotic digits test was also worse in the study group of another investigation with children diagnosed with dyslexia. 32 Likewise, in another study, the performance on the dichotic digits test was worse in the groups with dyslexia and attention deficit hyperactivity disorder (ADHD) when compared with the control group composed of children without previously diagnosed alterations. 31

Despite evaluating different auditory skills, both the PPS test and the Dichotic Digits test require full interhemispheric communication, so that the adequate transfer of auditory information and the execution of tasks can occur. Both tests were altered in TS, which may suggest a greater tendency to central alterations in this population. In the scientific literature, we have reports of alterations in cognition, learning, and even cases of intellectual disability in TS. Although we excluded participants previously diagnosed with these issues, we suggest that the central alterations in the auditory assessment may be related to the same cause of the other disorders mentioned.

Regarding the MLD test, in our study, we found a very similar score of alterations between the three age groups, ascertaining that about 1/3 of each group presented alterations in the test ( Table 2 ). Another Brazilian study evaluated MLD in 109 women, aged 20 to 30 years old, an age group like G3 in our research. In this study, an average threshold value of ∼ 10.83 dB was obtained, which is very similar to what we found in our G3, with an average threshold value of 11 dB. 28

Regarding the values found in children, in G1 of our study, in which girls aged 9 to 13 years old were evaluated, we found the average threshold value at 9.2 dB. This result corroborates a very recent study that evaluated the MLD in the age group from 7 to 12 years old, in which the threshold of 9.3 dB was suggested as a cutoff criterion. 33

Therefore, we can see that in the three groups of our research there was a greater number of participants with results within the normal range in the MLD test, which may suggest greater integrity of the auditory pathway in its initial portion, at the brainstem level.

The GIN test showed the same number of alterations in the MLD if we consider the total sample, with alterations in 13 of the 37 participants (35.1% of the sample). However, in the analysis of the results of the behavioral tests by age group, only the GIN test showed a statistically significant difference between the groups ( Table 2 ). The performance in this test was worse in the older age groups. This leads us to think of an issue related to the early aging of the central auditory system, as we have seen some reports in the literature mentioning early aging of peripheral hearing. 10 12 13

On the other hand, in an Egyptian study that evaluated 180 children and adolescents with normal hearing, no significant differences were observed in the performance of the GIN test in four age groups: I (6-8 years old ), II (> 8 to 10 years old), III (> 10 to 12 years old), IV (> 12 to 16 years old). 34 Regarding the total sample of our study, we observed changes in the GIN in just over 1/3 of the participants (35.1%). In the Brazilian survey that we found, the percentage of alterations observed in this test was 53.33% in children with reading and writing disabilities. 29

In terms of auditory maturation, we did not expect a difference relating to age group. According to the author of the test, 21 the performance observed in children from the age of 7 years old is like the performance in adults. 21 The confirmation that the participants understood the task proposed in the test was the fact that there were not many false-positive responses. This means that there was no response signaling at times when there was no silence gap, which indicates that the participants performed the task properly. 21

We observed the smallest number of alterations in the Filtered Speech Test. In this test, only 1 participant of the overall sample, belonging to G2 (14 to 19 years old), presented an altered result ( Table 2 ). In a study that evaluated the central auditory processing of children with reading and writing disabilities, only the filtered speech test presented a greater number of results within the normal range than altered results among six behavioral tests applied, and this corroborates our study data. 29

Like the MLD test, the Filtered Speech test is related to an initial portion of the central auditory pathway, also in the brainstem, which leads us to think of a correlation between TS with more central alterations, from the primary cerebral cortex to the interhemispheric connection in the corpus callosum area. We suggest new studies to correlate with the information found and the hypotheses raised in the present research.

Regarding the cytogenetic pattern, a greater number of alterations was observed in the participants with T1 cytogenetic pattern (monosomy X), in four of the five tests evaluated ( Table 3 ). The only test that showed a greater change in T2 was the filtered speech test, which was altered in only 1 participant (2.7% of the total sample). Therefore, this did not provide us with enough to infer that this cytogenetic pattern is associated with greater susceptibility to difficulties in auditory closure ability.

This greater number of alterations in the participants with monosomy X corroborates the studies that identify a greater number of auditory alterations in this cytogenetic pattern. 5 10 A study suggests an increased risk for hearing loss in the group with monosomy X when compared with other cytogenetic alterations observed in TS. 35

We did not find any studies that directly associate behavioral tests of central auditory processing in TS. We only found studies that correlate with peripheral auditory alterations, such as hearing loss and alterations in the middle ear. 10 35 In one of these studies, participants with monosomy X or isochromosome had hearing thresholds ∼ 10-11 dB worse than those with mosaicism or structural anomalies. 5 Another study suggests a greater propensity for middle ear problems in girls with monosomy X (45, X), in relation to those with mosaicism or deletion. 10

In the basic audiological assessment that we performed as inclusion and exclusion criteria for our study, we found hearing loss in 23 (35.3%) out of the 65 participants aged 9 to 39 years old who underwent pure-tone audiometry. 3 These data corroborate with another Brazilian study that identified hearing loss in 26% of the participants who underwent the exam, and with an Italian multicenter study, which found normal hearing in 45.7% of the 173 participants included in the study. 36

On the other hand, three other studies that performed audiometry in women with TS found many participants with hearing loss, where only 20% of the 112 ears evaluated in 1 study and only 17% of the 113 women evaluated in the other had normal hearing thresholds. And of the 213 children evaluated in the third study mentioned, 154 had hearing loss in at least 1 of the evaluated ears. 5 7

As a limitation of our study, we can mention the lack of correlation with neuropsychological assessment, with language tests and/or education assessment aiming to minimize issues related to the understanding of CAP tests. Despite that, we noticed that the participants understood the tests well, as they had no questions regarding their performance in the execution of the tasks.

We suggest further studies with the application of a battery of behavioral tests of central auditory processing in TS to better elucidate the issues raised in our study and promote adequate intervention for this population, such as auditory training directed to the training of altered auditory skills. 15

## Conclusion

We observed alterations in the auditory abilities of temporal ordering and temporal resolution, binaural interaction, and integration in the three age groups evaluated. The most impaired skills were temporal ordering, temporal resolution, and binaural integration. These skills were assessed by tasks that stimulate the auditory pathway at a more central level, involving the primary auditory cortex and interhemispheric connections, which leads us to think of how TS can interfere with the central auditory pathway, as well as affect other neurological issues.

Regarding the karyotype, a greater number of alterations was observed in the participants with T1 cytogenetic pattern (monosomy X), in four of the five assessed auditory skills (binaural integration, binaural interaction, temporal resolution, and temporal ordering). This cytogenetic pattern is the most cited as altered in other alterations, such as peripheral hearing loss and even cognitive and/or neurological issues.
